# Retrospective case series of peripheral neuropathy following carbon monoxide poisoning: clinical and electrophysiological characteristics

**DOI:** 10.1186/s12883-026-04830-8

**Published:** 2026-03-20

**Authors:** Zongyan Ma, Yanni Duan, Xingxing Huang, Yun Lang, Wen Li, Yujun Shi

**Affiliations:** 1 Department of Neurovascular Function Examination, The Second Hospital of Lanzhou University, Lanzhou, 730000 China; 2 The Second Hospital and Clinical Medical School, Lanzhou University, Lanzhou, 730000 China

**Keywords:** Carbon monoxide poisoning, Peripheral neuropathy, Nerve conduction, Electromyography

## Abstract

**Background:**

Peripheral neuropathy following carbon monoxide (CO) poisoning is considered a rare complication of CO intoxication. The following study analyzes the clinical features and electromyography (EMG) findings of patients with limb weakness after CO poisoning.

**Methods:**

A retrospective analysis was conducted on 13 patients from the Second Hospital of Lanzhou University database, diagnosed between January 2016 and November 2025. These patients presented with acute CO poisoning, concurrent limb weakness, and electrophysiologically confirmed peripheral neuropathy.

**Results:**

The mean age of the 13 patients with peripheral neuropathy after CO poisoning was 35.2 years (SD: 20.7, range 5–70). The severity of muscle weakness ranged from mild to complete paralysis. Electrophysiological testing revealed pure upper limb neuropathy (5/13), pure lower limb neuropathy (7/13), and combined upper and lower limb neuropathy (1/13). Among these cases, mononeuropathy was observed in 4/13, multiple mononeuropathy in 5/13, plexopathy in 3/13, and combined plexopathy with mononeuropathy in 1/13. Four patients exhibited pure motor nerve involvement, while one patient with multiple mononeuropathy showed pure motor nerve involvement in one affected nerve and combined sensorimotor nerve involvement in another. The remaining patients all demonstrated combined sensorimotor nerve involvement. Only two cases presented with combined myelin and axonal damage, while the others showed axonal damage.

**Conclusions:**

The significant limb weakness following CO poisoning is associated with peripheral neuropathy, which may manifest as mononeuropathy, multiple mononeuropathy, or plexopathy, primarily characterized by axonal damage.

**Supplementary Information:**

The online version contains supplementary material available at 10.1186/s12883-026-04830-8.

## Background

Carbon monoxide (CO) is produced by incomplete combustion of fossil fuels such as natural gas, liquefied petroleum gas, petroleum, wood, and coal [1]. CO poisoning constitutes a significant public health issue due to its high morbidity and mortality rates [2]. As CO is a colorless, odorless, and tasteless gas, CO exposure often goes unnoticed until symptoms appear [3]. This typically occurs due to malfunctioning residential heating systems or inadequate ventilation leading to incomplete coal combustion that releases CO into living spaces [4], and can also result from indoor charcoal grill usage in poorly ventilated areas [1]. In northern regions of China, it represents a frequently encountered emergency during winter and spring. CO poisoning affects the brain, heart, kidneys, skeletal muscles, skin, and peripheral nerves [5]. Depending on the exposure duration and CO concentration, patients may experience a wide spectrum of symptoms, ranging from headaches, fatigue, nausea, and dizziness to neuropsychological disorders, confusion, ataxia, seizures, loss of consciousness, cerebral or myocardial infarction, and even death [6]. Peripheral neuropathy is a rare complication of CO poisoning. To our knowledge, only one large-scale clinical study has specifically investigated CO-induced neuropathy [5], with most of the remaining literature consisting of case reports. In this context, we investigated the clinical and electrophysiological characteristics of 13 patients with peripheral neuropathy following CO poisoning, aiming to provide references for its diagnosis and prognosis.

## Methods

### Patients

Patients diagnosed with peripheral neuropathy following CO poisoning from January 2016 to November 2025 in the database of the Second Hospital of Lanzhou University were reviewed. The diagnosis of CO-induced peripheral neuropathy was based on the following criteria: 1) A clear history of CO exposure, with CO poisoning confirmed by detailed history, imaging, and laboratory tests; 2) Evidence of peripheral nerve damage determined by medical history, physical examination, and electrophysiological studies; 3) Confirmation by at least two neurologists of a temporal relationship between limb weakness and CO poisoning;4) Exclusion criteria included a history of compressive neuropathy, cervical or lumbar spine disease, diabetes, nutritional deficiencies, alcohol dependence, chemo/radiotherapy, rheumatologic/autoimmune disease, or familial/hereditary neuropathy. Muscle weakness was graded using the Medical Research Council (MRC) scale and categorized as: mild (MRC grade 4 or 4+), moderate (MRC grade 3), severe (MRC grade 2 or 1), or complete (MRC grade 0) [7, 8]. Recovery of muscle strength was defined as: 1) Complete recovery: muscle strength graded MRC 5 with resolution of sensory symptoms at final follow-up; 2) No recovery: no improvement in muscle strength; 3) Partial recovery: improvement falling between complete and no recovery. Additionally, all patients received approximately 10-minute follow-up assessments. The study was conducted in accordance with the Declaration of Helsinki and approved by the Human Ethics Committee of the Second Hospital of Lanzhou University (2025 A-1372). We confirm that written informed consent was obtained from all participants in the study. For all participants under the age of 16, written informed consent was obtained from their parents or legal guardians. For adult study subjects with cognitive impairment, written informed consent was provided by their legal guardians.

### Electrophysiology

Nerve conduction studies were performed using standard surface electrode techniques on a Nicolet Viking system (Wisconsin, USA) and a Keypoint electromyography machine (Dantec, Denmark). Skin temperature was maintained at ≥ 32.0 °C throughout the examination.

Motor nerve conduction studies assessed the following nerves: the median (wrist, elbow), ulnar (wrist, below elbow, above elbow), and radial (forearm, elbow, radial groove) nerves, all of which could receive additional supraclavicular fossa stimulation when indicated; as well as the tibial (ankle, popliteal fossa) and common peroneal (ankle, below fibular head, popliteal fossa) nerves. Recording muscles included the abductor pollicis brevis, abductor digiti minimi, extensor indicis/digitorum, abductor hallucis, and extensor digitorum brevis/tibialis anterior. For the musculocutaneous, axillary, and suprascapular nerves, stimulation at the supraclavicular fossa was performed with compound muscle action potentials (CMAPs) recorded from the biceps brachii, deltoid, and infraspinatus, respectively. Sensory nerve action potentials (SNAPs) were recorded orthodromically for the median, ulnar, and radial nerves, and antidromically for the superficial peroneal, saphenous, medial antebrachial cutaneous, and lateral antebrachial cutaneous nerves. The analyzed parameters included motor conduction velocity (MCV), distal motor latency (DML), and compound muscle action potential (CMAP) amplitude for motor studies, and sensory conduction velocity (SCV) and sensory nerve action potential (SNAP) amplitude for sensory studies. Needle electromyography (EMG) was performed using concentric needle electrodes to assess spontaneous activity, motor unit action potential duration, and recruitment patterns. All electrophysiological results were interpreted based on reference values established in our laboratory. Results were compared with the contralateral (asymptomatic) side or established normal values to determine the extent of injury. We assessed the presence of electrophysiological evidence of demyelination according to the criteria defined in the European Academy of Neurology/Peripheral Nerve Society (EAN/PNS) guidelines [9]. Axonal pathology was considered present if a side-to-side wave amplitude difference exceeded 50%, along with needle EMG findings of abnormal spontaneous activity, prolonged motor unit potential duration, or reduced recruitment.

## Results

### Patient demographics and clinical characteristics

Among the 13 patients with peripheral neuropathy following CO poisoning, the etiology included coal stove use in enclosed spaces (9 cases), a coal-heated bed (1 case), charcoal grilling (2 cases), and a natural gas malfunction during bathing (1 case). The mean age was 35.2 years (SD: 20.7; range: 5–70), with 9 males and 4 females. Eight patients presented with limb swelling. In 11 patients, the neuropathy occurred in limbs that had been subjected to prolonged pressure (on the same side) during coma. Muscle weakness severity ranged from mild to complete paralysis. Of the 13 patients, 8 underwent head imaging. Findings included abnormal signals involving bilateral cerebral white matter (centrum semiovale, periventricular), basal ganglia, and subcortical areas in 5 cases; bilateral basal ganglia lacunar infarcts in 1 case; and no abnormalities in 2 cases. During follow-up (ranging from 6 months to over 3 years in 13 cases), 2 patients achieved complete recovery within 6 months. Among the remaining patients, 8 showed partial improvement, and 3 had poor recovery, as detailed in Table 1.

**Table 1:** Demographic and clinical characteristics of 13 patients with peripheral neuropathy following carbon monoxide poisoning

Case	Sex	Age (years)	CO exposure time(hours)	Limb swelling	Pressure-dependent onset	Muscle weakness severity	Brain MRI	Cause of poisoning	Treatment	Follow-up time	Prognosis
1	Female	52	10 h	Yes	Yes	Severe	No	Coal stove	HBO, Neurotrophics, L imb rehabilitation	> 3 years	Partial recovery
2	Female	29	16 h	No	No	Mild	Multiple abnormal signal foci in bilateral frontal, parietal, temporal, occipital subcortical regions and bilateral basal ganglia	Coal stove	HBO, Corticosteroids, Neurotrophics, L imb rehabilitation	< 6 months	complete recovery
3	Male	6	10 h	Yes	Yes	Severe	Normal	Coal stove	HBO, Corticosteroids, Neurotrophics, L imb rehabilitation	> 2 years	Partial recovery
4	Male	64	24 h	Yes	Yes	Severe	Diffuse symmetrical abnormal signals in bilateral subcortical white matter, centrum semiovale, and periventricular regions	coal-heated bed	HBO, N eurotrophics, L imb rehabilitation	> 1 years	Partial recovery
5	Female	61	8 h	Yes	Yes	Severe	No	Faulty natural gas water heater	HBO	> 3 years	Partial recovery
6	Male	45	10 h	No	Yes	Severe	Multiple lacunar infarcts in the bilateral basal ganglia	Coal stove	HBO, Neurotrophics	< 1 years	Partial recovery
7	Male	21	10 h	Yes	Yes	Complete	No	Charcoal burning (barbecue)	HBO, N eurotrophics, L imb rehabilitation	> 2 years	Poor recovery
8	Male	30	12 h	No	Yes	Severe	No	Coal stove	HBO, N eurotrophics, L imb rehabilitation	> 3 years	Partial recovery
9	Male	36	10天	No	No	Mild	Patchy and linear abnormal signals in the bilateral centrum semiovale, periventricular white matter, and left frontal cortex, showing central softening in the white matter lesions	Coal stove	HBO, Corticosteroids, Neurotrophics, L imb rehabilitation	< 6 months	complete recovery
10	Male	70	10 h	Yes	Yes	Severe	No	Coal stove	HBO, N eurotrophics, L imb rehabilitation	> 3 years	Partial recovery
11	Male	16	11 h	Yes	Yes	Upper limb moderate, lower limb severe	Normal	Charcoal burning (barbecue)	HBO, N eurotrophics, L imb rehabilitation	> 6 months	Partial recovery in the upper limbs with poor recovery in the lower limbs
12	Male	5	11 h	Yes	Yes	Complete	Abnormalities are noted in the bilateral periatrial (occipital horn) and centrum semiovale white matter	Coal stove	HBO, N eurotrophics, L imb rehabilitation	> 6 months	Poor recovery
13	Female	12	12 h	No	Yes	Severe	A patchy abnormal signal focus in the right centrum semiovale	Coal stove	HBO, N eurotrophics, L imb rehabilitation	> 6 months	Partial recovery

*CO* carbon monoxide, *MRI*  Magnetic Resonance Imaging, *HBO* Hyperbaric Oxygen

### Electrophysiological characteristics

All patients underwent electrophysiological studies. Nerve conduction studies showed significantly reduced or absent compound muscle action potential (CMAP) and sensory nerve action potential (SNAP) amplitudes in affected nerves compared to the contralateral side or normal values. Nerve conduction velocities and distal motor latencies for all affected nerves were within normal limits (see Supplementary Material, Tables 4 and 5). Needle electromyography revealed varying degrees of spontaneous activity, prolonged motor unit potential (MUP) duration, and reduced recruitment in all affected muscles lesions (Tables 2 and 3). The distribution of lesions included pure upper limb neuropathy (5/13), pure lower limb neuropathy (7/13), and combined upper and lower limb neuropathy (1/13). The patterns of involvement were mononeuropathy (4/13), multiple mononeuropathy (5/13), plexopathy (3/13), and combined plexopathy with mononeuropathy (1/13). Four patients exhibited pure motor nerve involvement (Cases 2, 5, 6, 9). One patient with multiple mononeuropathy showed pure motor involvement in one nerve and combined sensorimotor involvement in another (Case 13). The remaining patients had combined sensorimotor involvement. Only two patients (Cases 3 and 11) exhibited combined demyelinating and axonal pathology, the demyelinating pattern manifested as conduction block. All other patients showed primarily axonal lesions (Tables 2 and 3).

**Table 2:** Electromyographic findings in patients with upper limb peripheral neuropathy following carbon monoxide poisoning

	4	5	6	10	11(first)	11(second)	13
CMAP of nerves (mV)/ MRC grade							
Median-APB							
wrist	6.01(26%↓)	10.9	10.9	7.3	8.6	8.69	0.07(99%↓)
elbow	5.13(37%↓)	10.9	10.4	4.9(27%↓)	8.2	8.15	0.09(99%↓)
supraclavicular fossa				4.2(37%↓)	0.75(91%↓)	8.10	
MRC grade	4+	5	5	4+	2	5	1
Ulnar-ADM							
wrist	NR	9.5	12.4	5.5(33%↓)	9.0	10.64	0.16(98%↓)
below elbow	NR	9.1	11.2	2.5(68%↓)	7.9	9.95	0.15(98%↓)
above elbow	NR	8.5	11.1	2.5(69%↓)	7.6	9.93	0.16(98%↓)
supraclavicular fossa	NR			2.7(66%↓)	0.89(91%↓)	9.79	
MRC grade	1	5	5	3	2	5	1
Radial-EDC							
elbow	NR	2.0(74%↓)	5.2(55%↓)	9.6	2.4(91%↓)	11.5	10.56
radial groove	NR	2.1(73%↓)	5.1(54%↓)	9.6			9.95
supraclavicular fossa	NR	2.0(76%↓)	3.1(72%↓)	9.1	0.90(92%↓)	11.3	9.95
MRC grade	1	2	3	5	2	5	5
Radial-EIP							
forearm	NR	3.0(42%↓)					4.65
elbow	NR	2.7(50%↓)					4.52
radial groove	NR						
MRC grade	1	2					5
Musculocutaneous- Biceps	14.08 (34%↓)			7.75	2.2(84%↓)	14	12.14
MRC grade	5			4	2	5	5
Axillary- Deltoid	8.29(50%↓)			8.1	2.4(80%↓)	12	11.36
MRC grade	4			4	2	5	5
Suprascapular- Infraspinatus	4.28(42%↓)			5.78			
MRC grade	5			5			
SNAP of nerves (µV)							
Median	3.8(51%↓)	15.0	5.8	NR	10.7	NR	NR
Ulnar	NR	8.9	6.0	NR	6.7	NR	9.2
Radial	NR	17.3	9.5	NR	16.7	NR	25.1
Medial antebrachial cutaneous	NR			NR	15.0	9.0(53%↓)	8.9
Lateral antebrachial cutaneous	20.1			NR	10.1	9.1(30%↓)	29.5
Needle electromyography							
Abnormal	Deltoid, E DC, F DI, APB	Triceps, E DC, EIP	EDC, EIP	Deltoid, B iceps, FCR, APB, FDI, FCU		FCR, EDC	APB, FDI, ADM
Normal	Infraspinatus, Biceps, L atissimus	FDI, APB, FCR, D eltoid, Biceps	Triceps, APB, FDI, B rachioradialis, Deltoid	Infraspinatus, Triceps, EDC		Infraspinatus, Deltoid, B iceps, Triceps, A PB, FDI	FPL, EIP, EDC, FCU, FCR, Biceps
Electrodiagnostic localization	Left brachial plexopathy involving the cords, p redominantly affecting the left radial and ulnar nerves( Axonal Injury)	Partial left radial neuropathy( Axonal Injury)	Partial right radial neuropathy( Axonal Injury)	Partial right brachial plexopathy ( Axonal Injury)	Partial left brachial plexopathy ( Predominantly demyelinating )	Partial left brachial plexopathy ( Predominantly demyelinating )	Severe right median and ulnar neuropathies ( Axonal Injury)

The amplitudes of CMAPs and SNAPs were compared with contralateral side; Needle electromyography is considered abnormal if it demonstrates spontaneous activity, or shows motor unit potentials with prolonged duration and reduced recruitment

*CMAP* Compound muscle action potential, *SNAP* Sensory nerve action potentia, *μV* Microvolt, *mV* Millivolt, *NR* No response, *APB* A bductor pollicis brevis, *ADM* Abductor digiti minimi, *EDC* Extensor digitorum communis, *EIP* Extensor indicis proprius, *FDI* First dorsal interosseous, *Biceps* Biceps brachii, *Latissimus* Latissimus dorsi, *Triceps* Triceps brachii, *FCR* Flexor carpi radialis, *FCU* Flexor carpi ulnaris, *FPL* Flexor pollicis longus

**Table 3:** Electromyographic findings in patients with lower limb peripheral neuropathy following carbon monoxide poisoning

	1	2	3	7	8	9	11(first)	11(second)	12
CMAP of nerves (mV) )/ MRC grade									
Peroneal - EDB									
ankle	NR	1.38 (86%↓)	NR	NR	1.69 (71%↓)	1.07(53%↓)/0.30(87%↓)	3.6 (61%↓)	NR	NR
below fibular head	NR	1.13 (86%↓)	NR	NR	1.38 (77%↓)	1.03(55%↓)/0.30(87%↓)	0.021(99%↓)	NR	NR
popliteal fossa	NR	1.13 (86%↓)	NR	NR					NR
MRC grade	1	4	1	0	2	4	2	1	0
Peroneal -TA									
below fibular head	0.85 (83%↓)	5.3	3.0 (40%↓)	NR		4.4/4.7	0.27 (95%↓)	NR	0.068(99%↓)
popliteal fossa	0.72 (86%↓)	5.1	1.26 (75%↓)	NR		4.4/4.6	0.27 (95%↓)	NR	NR
MRC grade	1	5	2	0		5	2	1	0
Tibial-AbH									
ankle	0.066(99%↓)	22.5	12.3 (37%↓)	0.069(99%↓)	6.2 (58%↓)	8.3/10.3	16.3	NR	NR
popliteal fossa	0.038(99%↓)	18.6	3.9 (75%↓)	0.057(99%↓)	5.4 (55%↓)	6.5/7.8	13.6	NR	
MRC grade	1	5	3	0	3	5	2	1	0
SNAP of nerves (µV)									
Sural	NR	38.1	NR	NR	NR	11.4/7.3	5.5 (39%↓)	NR	NR
Superficial peroneal	NR	17.1	10.1 (56%↓)	NR	NR	10.7/12.8	12.7	NR	NR
Saphenous	NR			2.7			4.9	5.1	
Needle electromyography									
Abnormal	VM, T A, G astroc, S T, G Max, G Med	EDB	TA, P L	TA, G astroc. BFsh, S T, G Max, G Med	TA, G astroc, BFsh, S T	EDB		TA, G astroc、BFsh、ST	TA、Gastroc
Normal		TA、PL、Gastroc、VM	BFsh、ST、Gastroc、TP、VM	VM、RF	GMax、GMed、VM	TA、PL、Gastroc、VM		GMax、GMed、VM	BFsh、ST、VM
Electrodiagnostic localization	Severe Right Lumbosacral Plexopathy( Axonal Injury)	Partial Injury to the Distal Right Deep Peroneal Nerve( Axonal Injury)	Combined Severe Left Common Peroneal ( Axonal Injury) and Partial Left Tibial Neuropathies(demyelination)	Severe Right Sciatic Nerve Injury with Mild Concomitant Injuries to the Right Superior and Inferior Gluteal Nerves( Axonal Injury)	Partial Left Sciatic Neuropathy( Axonal Injury)	Bilateral Distal Partial Deep Peroneal Neuropathies( Axonal Injury)	Partial Left Common Peroneal Neuropathy(Demyelination with Associated Axonal Loss)	Severe Left Sciatic Neuropathy( Axonal Injury)	Severe Right Common Peroneal and Tibial Neuropathies( Axonal Injury)

The amplitudes of CMAPs and SNAPs were compared with contralateral side; Needle electromyography is considered abnormal if it demonstrates spontaneous activity, or shows motor unit potentials with prolonged duration and reduced recruitment

*CMAP* Compound muscle action potential, *SNAP* Sensory nerve action potentia, *μV* Microvolt, *mV* Millivolt, *NR* No response, *EDB* E xtensor digitorum brevis, *TA* Tibialis anterior, *AbH* abductor hallucis, *VM* Vastus medialis, *Gastroc* Gastrocnemius, *PL* Peroneus longus, *ST* Semitendinosus, *BFsh* Biceps femoris (Short Head), *RF* Rectus femoris, *GMax* Gluteus maximus, *GMed* Gluteus medius, *TP* Tibialis posterior

### Descriptive cases

#### Case 4

A 64-year-old male patient was found unconscious by family members, unresponsive to verbal stimuli. He had slept on a coal-heated bed in a sealed room and was estimated to have been exposed to CO for approximately 24 h. Upon discovery, his left hip, lower leg, feet, and left upper arm exhibited skin redness and swelling, with visible blisters that had partially ruptured. He was urgently transferred to a local hospital. The arterial carboxyhemoglobin (COHb) was 12.4%, leading to the diagnoses of CO poisoning and pressure ulcers in the affected regions. After hyperbaric oxygen therapy, the patient gradually regained consciousness but was found to have weakness in the left upper limb, particularly in the left hand, presenting as wrist drop and finger drop. He reported numbness in the left ring finger, little finger, and thumb, as well as weakness in proximal limb lifting. Physical examination revealed proximal muscle strength graded 3 and distal muscle strength graded 0. Twenty-six days after onset, the patient gradually developed slowed responses and occasional urinary and fecal incontinence. The proximal weakness in the left upper limb improved slightly, but distal weakness remained significant.

Brain magnetic resonance imaging (MRI) revealed diffusely and symmetrically distributed abnormal signals in the subcortical regions of the bilateral frontal, parietal, temporal, and occipital lobes, as well as in the centrum semiovale and periventricular areas (see Supplementary Material). MRI plain scan of the unilateral humerus, u lna, radius, and adjacent joints showed diffuse abnormal signals in the long head, medial head, and lateral head of the left triceps brachii, brachialis muscle, and extensor carpi radialis longus and brevis muscles, predominantly affecting the upper arm muscle groups. There was extensive subcutaneous soft tissue exudation in the left upper arm and forearm, with partial fluid accumulation. EMG suggested Left brachial plexopathy involving the cords, predominantly affecting the left radial and ulnar nerves( Axonal Injury).

After six months of continued neurotrophic therapy and intensive limb rehabilitation, follow-up assessment revealed partial recovery of wrist dorsiflexion, though finger drop persisted and the patient regained functional grasping ability. The patient responsiveness mildly slower compared to pre-morbid levels, yet remained fully independent in activities of daily living.

#### Case 11

**Fig. 1:**
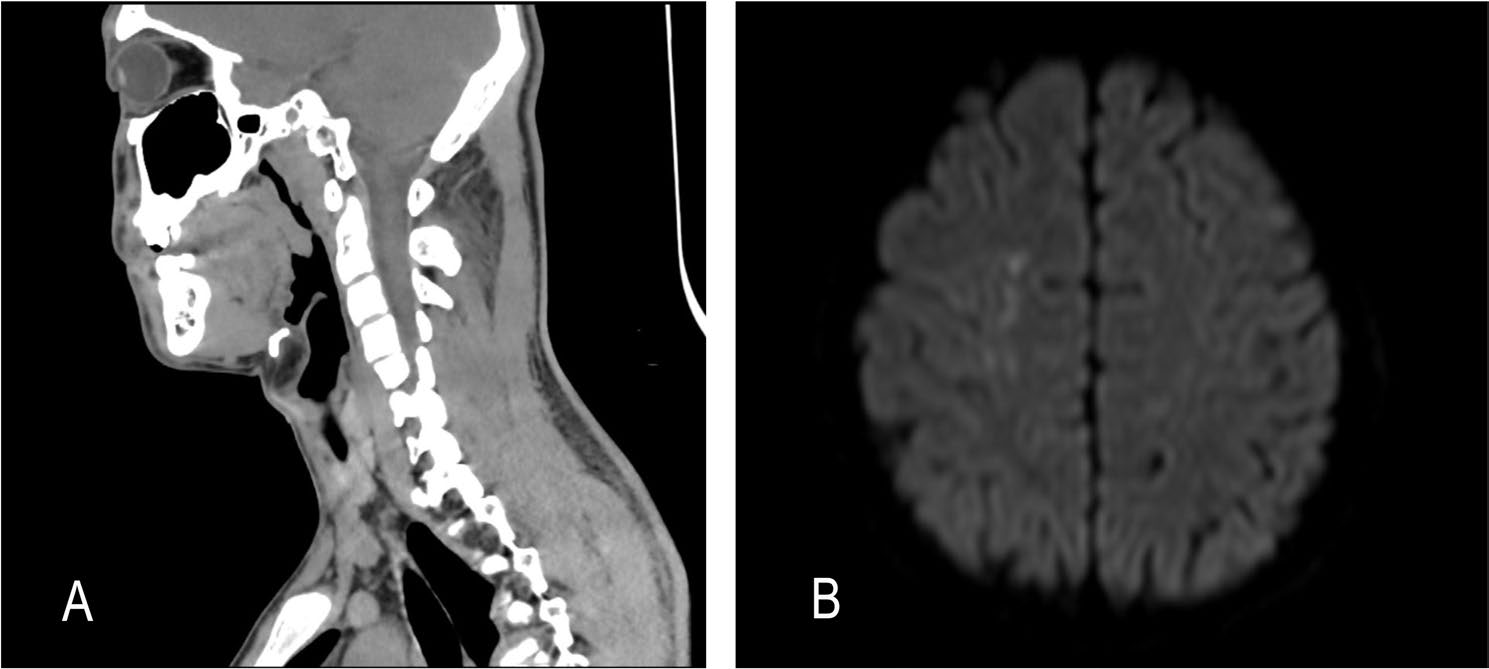
Neck CT revealed swelling of the left neck soft tissues, subcutaneous exudation, fluid accumulation, and mild swelling of the left parotid gland (**A**); Brain MRI upon admission showed patchy abnormal signal intensity in the right semioval center (**B**)

A 16-year-old male patient had a barbecue with his family using a charcoal grill in the evening. Before going to bed, the fire was not completely extinguished, and the doors and windows were closed. The patient was exposed to CO for approximately 11 h. When discovered by his family, he was in a state of confusion, with his left hip on the ground, his head and left upper limb leaning against the bedside, and had fecal incontinence. After being awakened, he reported dizziness, left neck pain, and pain and weakness in the left limbs. He was urgently sent to the emergency department of our hospital. Brain MRI showed no abnormalities; neck computed tomography (CT) revealed swelling of the left neck soft tissues, subcutaneous exudation, fluid accumulation, and mild swelling of the left parotid gland (Fig. 1A). Creatine kinase (CK) was > 50,000 U/L, and the arterial COHb was 8.6%. Physical examination showed muscle strength of grade 3 in both the proximal and distal left upper limb, grade 4 in the proximal left lower limb, and grade 1 in the distal left lower limb. The patient was diagnosed with CO poisoning, peripheral nerve damage, and rhabdomyolysis.

EMG performed four days after onset suggested left brachial plexus and common peroneal nerve damage (Demyelination with Associated Axonal Loss). Follow-up EMG six months later indicated left brachial plexus damage with significant improvement compared to previous findings, along with extremely severe left sciatic nerve damage (Axonal Injury). At the six-month follow-up, the patient showed normal cognitive responses, with the left upper limb having essentially returned to normal, whereas the left lower limb exhibited poor recovery. The patient’s first EMG examination was performed shortly after symptom onset, and needle EMG was not conducted. Based on the results of the two EMG examinations, it was considered that the upper limbs primarily exhibited demyelinating damage, which had largely recovered during follow-up. The patient presented with limb swelling. Initial physical examination revealed grade 4 muscle strength in the proximal lower limbs and grade 1 in the distal regions. However, follow-up EMG six months later showed extremely severe damage to the sciatic nerve. The possibility of ongoing damage persisting beyond four days after onset cannot be ruled out. An alternative explanation is that the full extent of axonal degeneration in the lower limb nerves was not yet complete at the time of the initial study, and thus the severity of the injury was only fully demonstrated on the subsequent examination. Due to the severe axonal pathology, the patient’s sciatic nerve damage showed poor recovery.

#### Case 12

A 5-year-old male patient was found by family members at noon the next day with impaired consciousness, accompanied by headache and vomiting. His grandmother and younger sister who were with him were in a coma. They had been using a coal stove and a kang (a traditional heated bed) for warmth in their home with doors and windows tightly closed, resulting in exposure to CO for approximately 10 h. Upon discovery, the patient presented with swelling in his legs, which were flexed and could not be extended, along with redness, swelling, and visible blisters on the skin of the right knee. He regained consciousness during transport to the local hospital but developed weakness in the right lower limb with limited extension, and dorsiflexion and plantar flexion muscle strength of the right foot were both grade 0. A brain MRI performed at the local hospital showed abnormal signals in the bilateral occipital horns of the lateral ventricles, centrum semiovale, and white matter regions. A lower extremity color Doppler ultrasound revealed swelling in the posterior muscles of the right calf, and an MRI scan of the right calf indicated abnormal signals in the tibialis anterior, gastrocnemius, soleus, peroneus longus, peroneus brevis muscles, intermuscular spaces, and subcutaneous soft tissues around the tibia. The CK level was 5584 U/L.

The patient was diagnosed with carbon monoxide poisoning, second-degree burns of the lower limbs, soft tissue injury in the lower limbs, and peripheral nerve injury. After hyperbaric oxygen therapy, the patient’s mental status returned to normal, with resolution of limb swelling and healing of the burned skin. However, weakness in the right lower limb persisted, prompting further evaluation at our hospital. EMG revealed severe axonal damage in the right common peroneal nerve and right tibial nerve. Following a course of symptomatic supportive treatment, including hyperbaric oxygen therapy, neurotrophic therapy, and limb rehabilitation, the patient showed improvement in knee extension, and the weakness in the right lower limb had partially resolved, allowing independent walking. However, dorsiflexion and plantar flexion of the right foot remained weak.

#### Case 13

A 12-year-old female patient was found unconscious alongside her mother in a rental home after her teacher, noticing her absence from school, contacted the landlord. They had used a coal stove and an electric blanket for heating before sleep, with the room’s doors and windows tightly closed, resulting in approximately 10 h of CO exposure. The patient’s mother died. Upon discovery, the patient presented with blisters on her right fingers, right cheek, and left elbow. She regained consciousness during emergency transport to the hospital but reported weakness in her right hand. Dorsiflexion was preserved; however, she was unable to extend her ring and little fingers, lift her thumb, and experienced numbness in the first three radial digits. Brain MRI upon admission showed patchy abnormal signal intensity in the right semioval center (Fig. 1B). Laboratory tests revealed a CK level of 11,963 U/L and the arterial COHb was 10.6%. Based on these findings, she was diagnosed with carbon monoxide poisoning, myocardial injury, rhabdomyolysis, and peripheral nerve injury.

After one month of symptomatic supportive treatment, including hyperbaric oxygen therapy, steroids, and neurotrophic therapy, her other symptoms improved, but weakness in the right hand persisted. Electromyography revealed severe injury to both sensory and motor fibers of the right median nerve and to the motor fibers of the right ulnar nerve at the wrist. Ultrasonography of the ulnar nerve demonstrated swelling of the right ulnar nerve at the wrist. The patient’s condition was primarily attributed to prolonged limb compression during coma, leading to median and ulnar nerve injury. Since the ulnar nerve injury occurred distal to Guyon’s canal after the divergence of the superficial and deep branches, the sensory fibers remainedintact. Following continued symptomatic supportive treatment—including hyperbaric oxygen therapy, hormone therapy, neurotrophic therapy, and limb rehabilitation—a six-month follow-up evaluation indicated partial clinical improvement. The patient regained the ability to grasp objects.

## Discussion

In this study, we found that peripheral neuropathy following carbon monoxide poisoning manifests as mononeuropathy, multiple mononeuropathy, or plexopathy. Electrophysiological studies are useful for assessing the extent and severity of nerve involvement. A large-scale clinical study by Choi IS examined 2,759 patients with acute carbon monoxide poisoning between 1976 and 1982, identifying 23 individuals (11 men and 12 women; mean age 29.3 years) with peripheral neuropathy confirmed by electrophysiological testing. Among these, 14 presented with sensory symptoms, 8 with mixed sensorimotor symptoms, and only 1 with pure motor symptoms. The lower extremities were affected in most cases, with only two cases involving other sites. That study also indicated that peripheral neuropathy after carbon monoxide poisoning predominantly affects young individuals, and all patients recovered within 3 to 6 months [5]. In contrast to those findings, our case series showed involvement of both upper and lower extremities, including isolated upper limb neuropathy (5/13), isolated lower limb neuropathy (7/13), and combined upper and lower limb neuropathy (1/13), with no significant difference observed. Only two patients returned to normal within 6 months; the remaining patients showed partial or poor recovery during follow-up periods exceeding 6 months, resulting in long-term residual neurological deficits. Eight patients had simultaneous involvement of sensory and motor fibers, four had pure motor fiber involvement, and one patient with combined median and ulnar nerve lesions had median nerve lesions affecting both motor and sensory fibers while the ulnar nerve lesions affected only motor fibers. Among the four cases with pure motor fiber involvement, one presented with bilateral deep peroneal nerve lesions, and one with unilateral deep peroneal nerve lesions. The remaining two patients had radial nerve lesions without sensory fiber involvement: Case 5 had no sensory symptoms, whereas Case 6 reported sensory symptoms. All other patients exhibited combined sensory and motor nerve involvement, and no cases of pure sensory nerve involvement were observed. Only two patients showed mixed demyelinating and axonal lesions, with myelin damage primarily manifesting as proximal conduction block; the remaining patients had axonal lesions alone.

A review of the literature indicates that peripheral neuropathy following carbon monoxide poisoning has been primarily documented in case reports, with manifestations including unilateral brachial plexus [10, 11], bilateral brachial plexus [12], sciatic nerve [13, 14], bilateral symmetric ulnar nerve [15], bilateral facial nerve [15], optic nerve [16], diaphragmatic paralysis [17], and multiple sensorimotor neuropathies [18]. The cases we reported included mononeuropathy, multiple mononeuropathies, and plexopathy, involving the median, ulnar, radial, common peroneal, tibial, and sciatic nerves, as well as the brachial plexus and lumbosacral plexus. These findings highlight the diverse presentations of peripheral neuropathy induced by carbon monoxide poisoning and underscore the need for careful differentiation in the diagnostic process.

Studies by Choi IS, García A, et al. demonstrated demyelination on peripheral nerve biopsies and demyelination with axonal preservation on electrophysiological studies following carbon monoxide poisoning [19, 20]. Rahmani M, et al. reported bilateral brachial plexus injuries and suggested a favorable prognosis for peripheral neuropathy after CO poisoning [12]. However, some studies have also reported prolonged and poor recovery of neuropathy following carbon monoxide poisoning. In these cases, abnormal spontaneous activity was observed on needle electromyography, suggesting that when peripheral neuropathy after CO poisoning presents as axonal damage, the recovery period is significantly extended [10, 11, 13]. In the present case series, electrophysiological examinations revealed that two patients had concomitant demyelinating and axonal damage, while the remaining patients showed either low or absent amplitudes of compound muscle action potentials (CMAPs) or sensory nerve action potentials (SNAPs). Needle electromyography demonstrated spontaneous activity or prolonged motor unit potential (MUP) duration with reduced recruitment, indicating that the nerve damage was predominantly characterized by motor and sensory axonal neuropathy. The severity of neural impairment influenced patient prognosis, resulting in recovery processes of varying durations.

Regarding the mechanism of peripheral nerve injury secondary to carbon monoxide poisoning, several hypotheses have been proposed. In a series by Choi IS, all 20 patients with localized swelling presented with peripheral neuropathy, and among 23 patients with peripheral neuropathy, 19 had localized swelling in the affected areas. These findings suggest that pressure effects from localized swelling may be a significant contributing factor to the development of neuropathy [5]. A review of the literature indicates that the proposed mechanisms of peripheral nerve injury in reported cases include local edema, direct nerve compression, CO cytotoxicity, hypoxic-ischemic injury, punctate hemorrhage, edema secondary to venous obstruction, circulatory disturbances in comatose patients, and soft tissue necrosis [10–13, 15, 19, 21, 22].

In the present case series, 11 patients had a history of prolonged compression on the affected limb, and 8 had a history of localized limb swelling. These observations support the role of direct nerve compression and pressure effects from local swelling as important contributors to nerve injury. The literature further indicates that compressive neuropathies tend to occur at anatomically predisposed sites, including the median nerve at the wrist, the ulnar nerve at the elbow, the radial nerve (at the radial groove and supinator muscle), and in the lower limbs, the common peroneal nerve at the fibular head and the tibial nerve at the tarsal tunnel. These lesions typically localize to anatomically predisposed sites—such as fascial, muscular, or osseous tunnels near joints—and are frequently associated with occupation- or sports-related repetitive strain injuries. They may also arise perioperatively, result from suboptimal intensive care management, or stem from specific habits (e.g., prolonged leg crossing, sleeping on an arm, or squatting) that lead to acute compressive neuropathies. During compression, patients often present with pronounced numbness and weakness in the affected limb, sometimes accompanied by pain. Notably, such patients have a clear history of compression but no evidence of limb swelling. Electrophysiological findings typically reveal focal demyelination, including prolonged distal latency, slowed conduction velocity, and conduction block. These patients generally have a short recovery period and a favorable prognosis. However, recovery may be prolonged if axonal damage occurs, requiring collateral sprouting and axonal regeneration. Few patients present with pure axonal damage [23–29]. In the present case series, axonal damage was the predominant finding, with only two patients exhibiting conduction block. Importantly, these cases were also associated with limb swelling, making pure compressive neuropathy secondary to carbon monoxide poisoning less likely in this cohort. Among the five patients without a history of limb swelling who predominantly presented with axonal damage, the following observations were made. In Case 6, no significant bilateral difference was observed in the superficial radial nerves; however, the patient reported numbness in the radial aspect of the hand dorsum, suggesting injury at the upper arm level. Case 13, a pediatric patient, developed right hand weakness and numbness in the three radial fingers upon awakening during transport after loss of consciousness due to CO poisoning, with no prior history of hand numbness or weakness. Needle electromyography revealed abnormalities localized to the hand muscles innervated by the median and ulnar nerves, while proximally innervated muscles were spared. Combined with ulnar nerve ultrasound findings, distal median and ulnar nerve damage was suspected, with ulnar nerve injury likely occurring distal to Guyon’s canal, after the division of its superficial and deep branches (sparing sensory fibers). Both cases involved common entrapment sites. Case 8 involved sciatic nerve injury without prior limb numbness, weakness, or trauma. Cases 2 and 9, both with deep peroneal nerve involvement, had prolonged loss of consciousness; one of these patients also sustained heel burns. While secondary compressive neuropathy due to CO poisoning and posture-related neuropathy were considered likely contributors, electrophysiological findings alone cannot definitively distinguish these entities from direct CO-induced nerve injury. Among the eight reported cases, all had a history of localized limb swelling and prolonged compression of the affected limb with neurological deficits, with only a minority of injury sites localized to typical compression points. This suggests that peripheral neuropathy following carbon monoxide poisoning is not solely attributable to compressive mechanisms, and primary neuropathy related to CO poisoning should also be considered. Unfortunately, electrophysiological studies are unable to differentiate between these potential mechanisms. In summary, we propose that limb swelling and direct compression may represent important contributing factors in the development of peripheral neuropathy after carbon monoxide poisoning. It is likely that both primary CO-related neuropathy and secondary compressive neuropathy coexist. The hypoxia induced by CO, along with subsequent ischemia, punctate hemorrhage, the direct cytotoxic effects of CO itself, circulatory disturbances, and soft tissue necrosis, may collectively contribute to neural damage in affected patients. Attributing the pathological mechanism solely to any one of the aforementioned hypotheses is difficult; therefore, a multifactorial etiology appears more plausible.

This study has several limitations, including the small sample size of rare delayed peripheral neuropathy cases following carbon monoxide poisoning, its single-center design, and its retrospective nature. Data collection was incomplete for some patients, with a lack of follow-up electromyography results. Additionally, certain cases may require longer follow-up periods for comprehensive prognostic evaluation. Pressure-induced paralysis resulting from prolonged coma constitutes a confounding factor that was difficult to completely eliminate in this study.

## Conclusions

In summary, this study highlights the clinical and electrophysiological features of this rare form of peripheral neuropathy following CO poisoning. The pattern of nerve involvement may manifest as mononeuropathy, multiple mononeuropathies, or plexopathies, accompanied by significant limb weakness. This can include damage to individual nerves such as the median, ulnar, radial, peroneal, tibial, and sciatic nerves, as well as injuries to the brachial and lumbosacral plexuses. This illustrates the diverse clinical manifestations of peripheral neuropathy induced by CO poisoning, which should be carefully considered during differential diagnosis. Predominantly axonal neuropathy was observed, characterized by prolonged recovery periods that can severely impact patients’ quality of life. This information is essential for neurologists to avoid missed diagnoses and ensure timely intervention within the optimal treatment window. Additionally, in patients presenting with limb weakness or bradykinesia, peripheral nerve injury should be considered in the differential diagnosis. Electromyography is valuable for assessing the extent and severity of nerve damage, as well as for prognostic evaluation. However, the exact pathogenic mechanism of peripheral neuropathy after CO poisoning remains unclear. Further multicenter prospective investigations and pathophysiological studies on peripheral neuropathy following carbon monoxide poisoning are needed to clarify its underlying mechanisms, enabling the development of personalized diagnostic and treatment strategies to improve patient outcomes and enhance their quality of life.

## Supplementary Information

Supplementary Material 1.

Supplementary Material 2.

Supplementary Material 3.

## Data Availability

No datasets were generated or analysed during the current study.

## Abbreviations

CO: Carbon monoxide
EMG: Electromyography
MRC: Medical research council
CMAPs: Compound muscle action potentials
SNAPs: Sensory nerve action potentials
MCV: Motor conduction velocity
DML: Distal motor latency
SCV: Sensory conduction velocity
NCV: Nerve conduction velocity
MUP: Motor unit potential
COHb: Carboxyhemoglobin
MRI: Magnetic resonance imaging
CT: Computed tomography
CK: Creatine kinase
HBO: Hyperbaric oxygen
µV: Microvolt
mV: Millivolt
NR: No response
APB: Abductor pollicis brevis
ADM: Abductor digiti minimi
EDC: Extensor digitorum communis
EIP: Extensor indicis proprius
FDI: First dorsal interosseous
FCR: Flexor carpi radialis
FCU: Flexor carpi ulnaris
FPL: Flexor pollicis longus
EDB: Extensor digitorum brevis
TA: Tibialis anterior
AbH: Abductor hallucis
VM: Vastus medialis
PL: Peroneus longus
ST: Semitendinosus
BFsh: Biceps femoris (Short Head)
RF: Rectus femoris
GMax: Gluteus maximus
GMed: Gluteus medius
TP: Tibialis posterior
