# Supplementary material for: Retrospective case series of peripheral neuropathy following carbon monoxide poisoning: clinical and electrophysiological characteristics
Source: BMC Neurol. 2026 Mar 20;26:285. doi: 10.1186/s12883-026-04830-8 (PMC13126964; doi:10.1186/s12883-026-04830-8)
Supplement: Supplementary file 1 — Supplementary Material 1. [file 12883_2026_4830_MOESM1_ESM.docx]

**Table 4** Electromyographic findings in patients with upper limb peripheral neuropathy following carbon monoxide poisoning

|  | 4 | 5 | 6 | 10 | 11(first) | 11(second) | 13 |
| --- | --- | --- | --- | --- | --- | --- | --- |
| **DL(ms)/MCV****(m/s)** | | | | | | | |
| Median-APB |  |  |  |  |  |  |  |
| wrist | 2.60 | 3.02 | 3.39 | 4.15 | 2.94 | 3.18 | 4.21 |
| elbow | 5.78/61 | 5.63/77 | 7.29/54 | 9.25/54.1 | 6.54/54.2 | 7.34/50 | 9.33/58 |
| supraclavicular fossa |  |  |  | 16.2/56.8 | 12.4 | 13.5 |  |
| Ulnar-ADM |  |  |  |  |  |  |  |
| wrist | NR | 2.71 | 2.55 | 3.05 | 2.27 | 2.45 | 2.71 |
| below elbow | NR | 5.10/67 | 5.16/69 | 7.82/50.3 | 4.71/69.7 | 5.52/56 | 5.57/58 |
| above elbow | NR | 7.29/48 | 7.71/50 | 10.5/49.8 | 6.38/50.9 | 7.29/51 | 6.88/53 |
| supraclavicular fossa | NR | 8.85/51 | 9.32/65 | 17.0/52.7 | 12.6 | 12.71 |  |
| Radial-EDC |  |  |  |  |  |  |  |
| elbow | NR | 2.19 | 1.88 | 2.90 | 2.09 | 2.11 | 1.61 |
| radial groove | NR | 3.96/78 | 3.85/61 | 4.67/62.1 |  |  | 3.18/64 |
| supraclavicular fossa | NR | 6.72 | 8.65/63 | 8.29/77.3 | 6.99 | 7.02 | 5.94 |
| Radial-EIP |  |  |  |  |  |  |  |
| forearm | NR | 3.85 |  |  |  |  | 2.34 |
| elbow | NR | 6.04/64 |  |  |  |  | 3.91/67 |
| radial groove | NR |  |  |  |  |  |  |
| Musculocutaneous- Biceps | 1.93 |  |  | 4.75 | 2.27 | 2.26 | 3.59 |
| Axillary- Deltoid | 2.24 |  |  | 4.15 | 2.06 | 2.05 | 2.55 |
| Suprascapular- Infraspinatus | 3.17 |  |  | 2.85 |  |  |  |
| **SCV (m/s)** | | | | | | | |
| Median | 58 | 53 | 53 | NR | 60.3 | NR | NR |
| Ulnar | NR | 60 | 48 | NR | 55.6 | NR | 52 |
| Radial | NR | 51 | 59 | NR | 68.0 | NR | 51 |
| Medial antebrachial cutaneous | NR |  |  | NR | 58 | 59 | 62 |
| Lateral antebrachial cutaneous | 60 |  |  | NR | 51 | 50 | 54 |

Motor nerve conduction velocity(MCV),Distal latency(DL), Sensory nerve conduction velocity (SCV), No response(NR), Abductor pollicis brevis(APB), Abductor digiti minimi(ADM), Extensor digitorum communis（EDC）, Extensor indicis proprius（EIP）
