# Supplementary material for: Retrospective case series of peripheral neuropathy following carbon monoxide poisoning: clinical and electrophysiological characteristics
Source: BMC Neurol. 2026 Mar 20;26:285. doi: 10.1186/s12883-026-04830-8 (PMC13126964; doi:10.1186/s12883-026-04830-8)
Supplement: Supplementary file 2 — Supplementary Material 2. [file 12883_2026_4830_MOESM2_ESM.docx]

**Table 5** Electromyographic findings in patients with lower limb peripheral neuropathy following carbon monoxide poisoning

|  | 1 | 2 | 3 | 7 | 8 | 9 | 11 (first) | 11(second) | 12 |
| --- | --- | --- | --- | --- | --- | --- | --- | --- | --- |
| **DL(ms)/MCV****(m/s)** | | | | | | | | | |
| Peroneal - EDB |  |  |  |  |  |  |  |  |  |
| ankle | NR | 4.46 | NR | NR | 4.20 | 4.36/4.68 | 3.56 | NR | NR |
| below fibular head | NR | 10.5/44.7 | NR | NR | 11.9/40.0 | (9.98/48.0)/( 10.0/50.8) | 13.4/40.7 | NR | NR |
| popliteal fossa | NR | 12.6/46.1 | NR | NR |  |  |  |  | NR |
| Peroneal -TA |  |  |  |  |  |  |  |  |  |
| below fibular head | 3.96 | 3.47 | 1.98 | NR |  | 3.57/3.60 | 3.96 | NR | 4.36 |
| popliteal fossa | 6.60/41.7 | 6.07/45.2 | 3.13/65 | NR |  | (6.56/49.1)/( 6.61/42.8) | 6.57/42.0 | NR | NR |
| Tibial-AbH |  |  |  |  |  |  |  |  |  |
| ankle | 3.88 | 4.55 | 3.19 | 5.47 | 4.17 | 3.17/3.33 | 3.52 | NR | NR |
| popliteal fossa | 12.1/45.0 | 11.7/49.7 | 8.38/44.3 | 14.7/41.2 | 13.9/40.5 | (9.63/51.7)/(9.98/49.6) | 10.8/50.8 | NR |  |
| **SCV (m/s)** | | | | | | | | | |
| Sural | NR | 42.6 | NR | NR | NR | 56.5/52.8 | 48.1 | NR | NR |
| Superficial peroneal | NR | 43.3 | 46.4 | NR | NR | 50.0/55.8 | 52.9 | NR | NR |
| Saphenous | NR |  |  | 41.4 |  |  | 42 | 42 |  |

Motor nerve conduction velocity(MCV),Distal latency(DL), Sensory nerve conduction velocity (SCV), No response(NR) , Extensor digitorum brevis (EDB), Tibialis anterior(TA), abductor hallucis (AbH)
