# Supplementary figures and images for: Retrospective case series of peripheral neuropathy following carbon monoxide poisoning: clinical and electrophysiological characteristics
Source: BMC Neurol. 2026 Mar 20;26:285. doi: 10.1186/s12883-026-04830-8 (PMC13126964; doi:10.1186/s12883-026-04830-8)

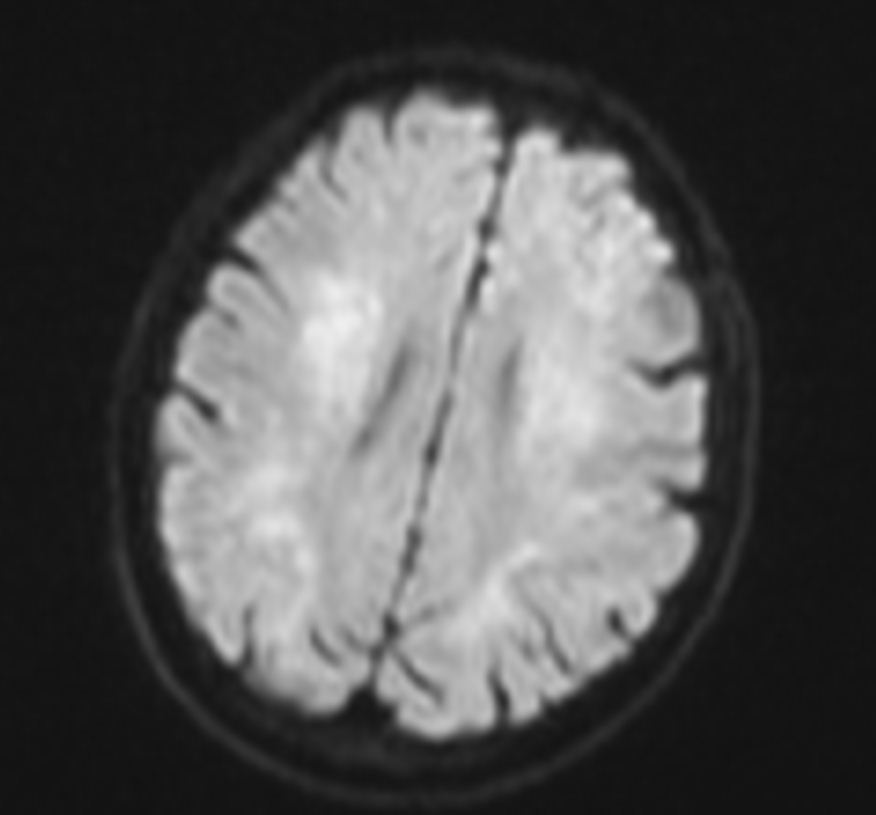

Supplement: Supplementary file 3 — Supplementary Material 3. [file 12883_2026_4830_MOESM3_ESM.tif]
